# Shedding Light on the Shadows: A Trio of Neuro-Ophthalmic Cases Emerging From Rapid Weight Loss-Induced Thiamine Deficiency

**DOI:** 10.7759/cureus.81648

**Published:** 2025-04-03

**Authors:** Hesham Eissa, Khalid Elhassan, Amani Alzaabi, Mohamed E Abouelnaga, Mahfoud Elbashari

**Affiliations:** 1 Neurology-Internal Medicine, Zayed Military Hospital, Abu Dhabi, ARE

**Keywords:** blurring of vision, neuro-ophthalmic manifestations, rapid weight loss, strenuous exercise, thiamine deficiency

## Abstract

Thiamine (vitamin B1), a critical cofactor for carbohydrate metabolism and neurotransmitter synthesis, plays an indispensable role in neuro-ophthalmic health. Deficiency of this crucial nutrient, while traditionally associated with a range of neuro-ophthalmic symptoms, can often lead to clinical presentations that are far from typical. In this case series, we delve into the unique experiences of three young men who developed uncommon neuro-ophthalmic manifestations due to thiamine deficiency following rapid weight loss due to strenuous exercises. Involvement of the optic nerve, presenting as either optic neuritis or papilledema, stands as an unusual revelation of thiamine deficiency; the insidious nature of imaging abnormalities, which require time to manifest, poses an additional diagnostic challenge. It is crucial to recognize that factors such as rapid weight loss and strenuous exercise can precipitate thiamine deficiency, potentially hastening the onset of its neuro-ophthalmic manifestations. Prompt recognition and intervention are essential to mitigate the risk of permanent neurologic deficits, emphasizing the imperative need for awareness and vigilance among healthcare practitioners. Through this series, we aim to augment the understanding of this under-recognized clinical entity.

## Introduction

Thiamine (vitamin B1) is required for the activity of numerous enzymes such as transketolase, alpha-ketoglutarate dehydrogenase, pyruvate dehydrogenase, and branched-chain amino acid dehydrogenase. Thiamine is a water-soluble vitamin that plays an important role in carbohydrate metabolism and thus adenosine triphosphate (ATP) production. Thiamine is also needed to produce neurotransmitters and the myelin sheath [1]. The central nervous system is almost completely dependent on glucose metabolism for energy and is therefore susceptible to thiamine deficiency [1]. Thiamine deficiency can cause vague symptoms, making diagnosis difficult. Wernicke's encephalopathy includes neuro-ophthalmic manifestations of thiamine deficiency [2]. Ocular manifestations like ophthalmoplegia and nystagmus were described as a hallmark of the disorder, affecting about 85% of patients. However, only 15% to 30% present with the complete triad of Wernicke's encephalopathy, including ophthalmoplegia, ataxia, and confusion [2]. Thiamine deficiency can be broadly divided into alcoholic and non-alcoholic based on the etiologic factors. Thiamine deficiency is common in critically ill patients and those with high metabolic demands like pregnancy and hyperthyroidism [1]. Thiamine deficiency can also be caused by persistent vomiting, like in patients with hyperemesis gravidarum, drug-induced hyperlactataemia, and an acute gastrointestinal illness in already malnourished individuals [3]. Thiamine is absorbed in the jejunum. The body stores about 30 mg of thiamine, which is relatively small, mostly in the brain, liver, kidney, heart, and skeletal muscles. The recommended dietary allowance of thiamine for adults is 1.1-1.2 mg/day [1, 4]. Thiamine is present in many animal and plant-based products, such as yeast, whole grains, legumes, and pork, but it is deficient in fats, oils, and refined sugars. Highly processed, high-carbohydrate, low-calorie, and unbalanced weight regimens are common dietary patterns that lead to thiamine deficiency [4, 5]. Chronic alcoholics and people who have had gastric bypass surgery are at the highest risk of thiamine deficiency. Thiamine is converted to its active form, thiamine triphosphate (TTP), by the liver after it is absorbed from the gut. Alcoholism can cause hepatic cirrhosis, which can lead to thiamine deficiency symptoms [6]. We present a case series of three young men with neuro-ophthalmic symptoms of thiamine deficiency.

## Case presentation

Case 1

A 17-year-old male patient presented with a three-week history of vomiting, epigastric and right upper quadrant pain, and dizziness. Over the past 15 months, he had lost 30 kg as he went on an unbalanced weight-loss regimen with very low calories and consumed a high-carbohydrate diet. The weight loss and dietary patterns coincided with his recent initiation into strenuous exercise routines. He described the dizziness as being present even while lying down, associated with double vision, and the symptoms were exacerbated when looking to the sides. The patient denied any use of illicit drugs or alcohol. Upon examination, the patient displayed bidirectional horizontal nystagmus on end gaze. The fundoscopy examination was normal. No gait abnormalities were observed. Romberg's sign was negative. The rest of the neurological exam was unremarkable.

The patient underwent a comprehensive workup to investigate the symptoms and underlying causes of their condition. Blood tests revealed normal white blood cells and platelet counts, indicating no immediate signs of infection or significant inflammation. However, the C-reactive protein (CRP) level was elevated, suggesting some degree of inflammation. Bilirubin levels were initially high, including total bilirubin and direct bilirubin, but both improved, indicating a resolving condition affecting the liver or biliary tract. Potassium levels were slightly low. Liver function tests, including aspartate transaminase (AST) and alanine aminotransferase (ALT), were normal, as were alkaline phosphatase (ALP) levels.

Critical to the diagnosis, the patient's thiamine levels were significantly below the normal range, coupled with low transketolase activity. A TPP effect was observed to be more than 90 ng/ml, highlighting a significant thiamine deficiency. Urinalysis showed the presence of ketones (3+), bilirubin (+3), and trace amounts of blood, with 11 to 20 white blood cells per high power field (WBC/HPF), though a repeat test was negative for nitrites and leukocytes, suggesting transient urinary tract irritation or infection. Additionally, testing for aquaporin-4 antibodies (AQP4-Ab), which are indicative of neuromyelitis optica spectrum (NMOSD), returned negative. The patient's laboratory results are summarized in Table 1.

**Table 1:** A summary of Case 1's laboratory results CRP: C-reactive protein; TPP: thiamine pyrophosphate effect mg/dL (milligrams per deciliter), mmol/L (millimoles per liter), ng/L (nanograms per liter), U/L (units per liter), and ng/mL (nanograms per milliliter) are standard measurement units.

Test	Result	Normal range	Deviation
CRP	24 mg/dL	< 0.3 mg/dL	High
Total bilirubin	71 mg/dL → 39 mg/dL	0.1 - 1.2 mg/dL	High
Direct bilirubin	34 mg/dL → 22 mg/dL	< 0.3 mg/dL	High
Potassium	3.3 mmol/L	3.6 - 5.2 mmol/L	Low
Thiamine (vitamin B1)	15 ng/L	70 - 180 ng/L	Low
Transketolase activity	18.4 U/L	42 - 100 U/L	Low
TPP effect	> 90 ng/mL	< 20 ng/mL	High

**Figure 1:**
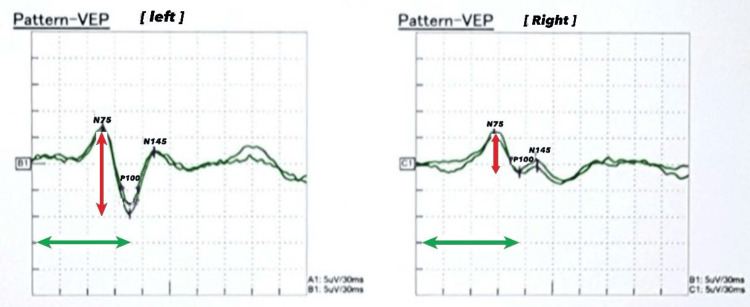
The VEP of Case 1 shows mildly prolonged P100 latency and reduced amplitude from the left eye as compared to the right side, denoting mild conduction defects in the anterior visual pathway of the left optic nerve. VEP: visual evoked potential

**Figure 2:**
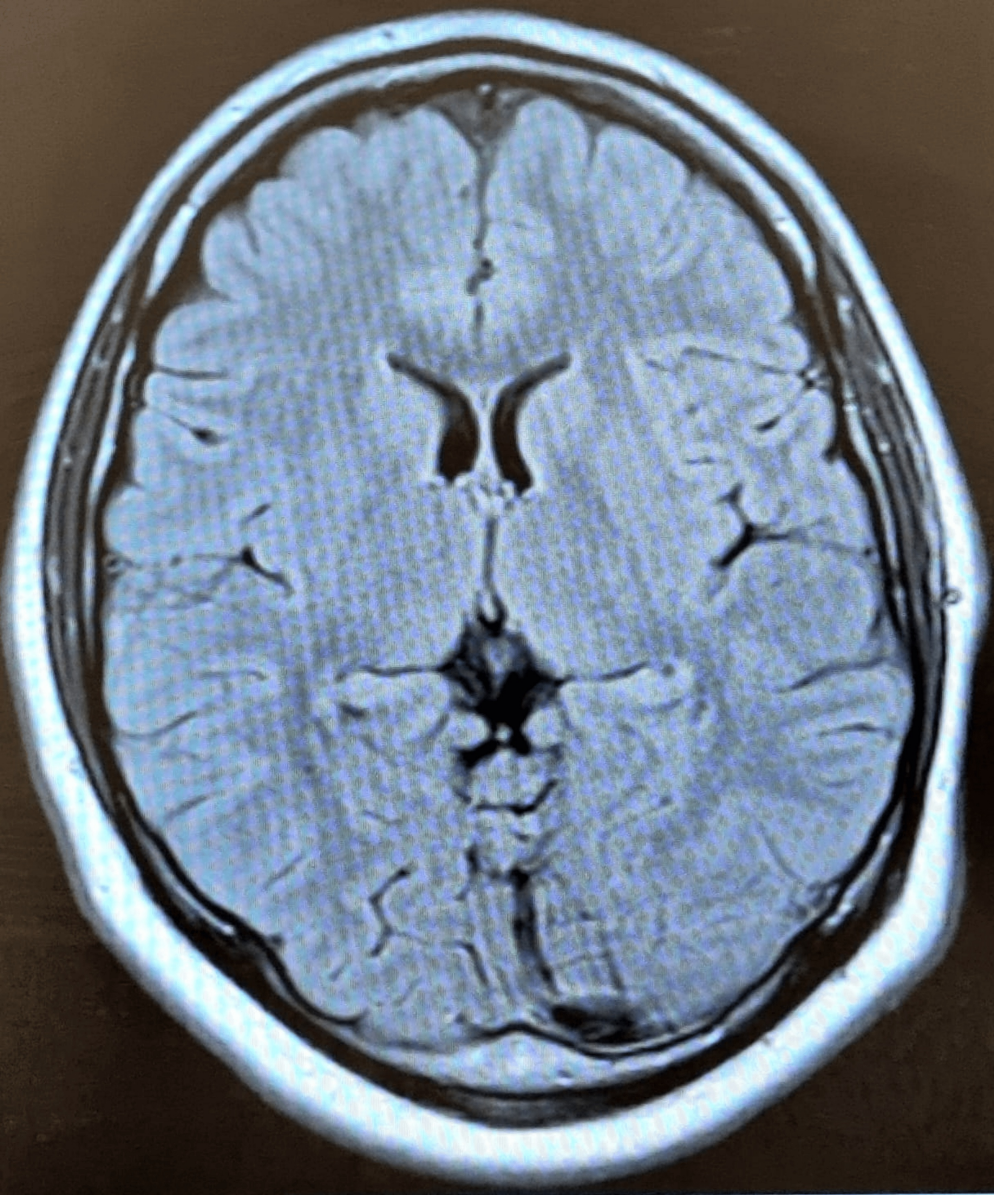
The MRI of Case 1's brain (axial FLAIR image) shows no gross pathology. FLAIR: fluid-attenuated inversion recovery

**Figure 3:**
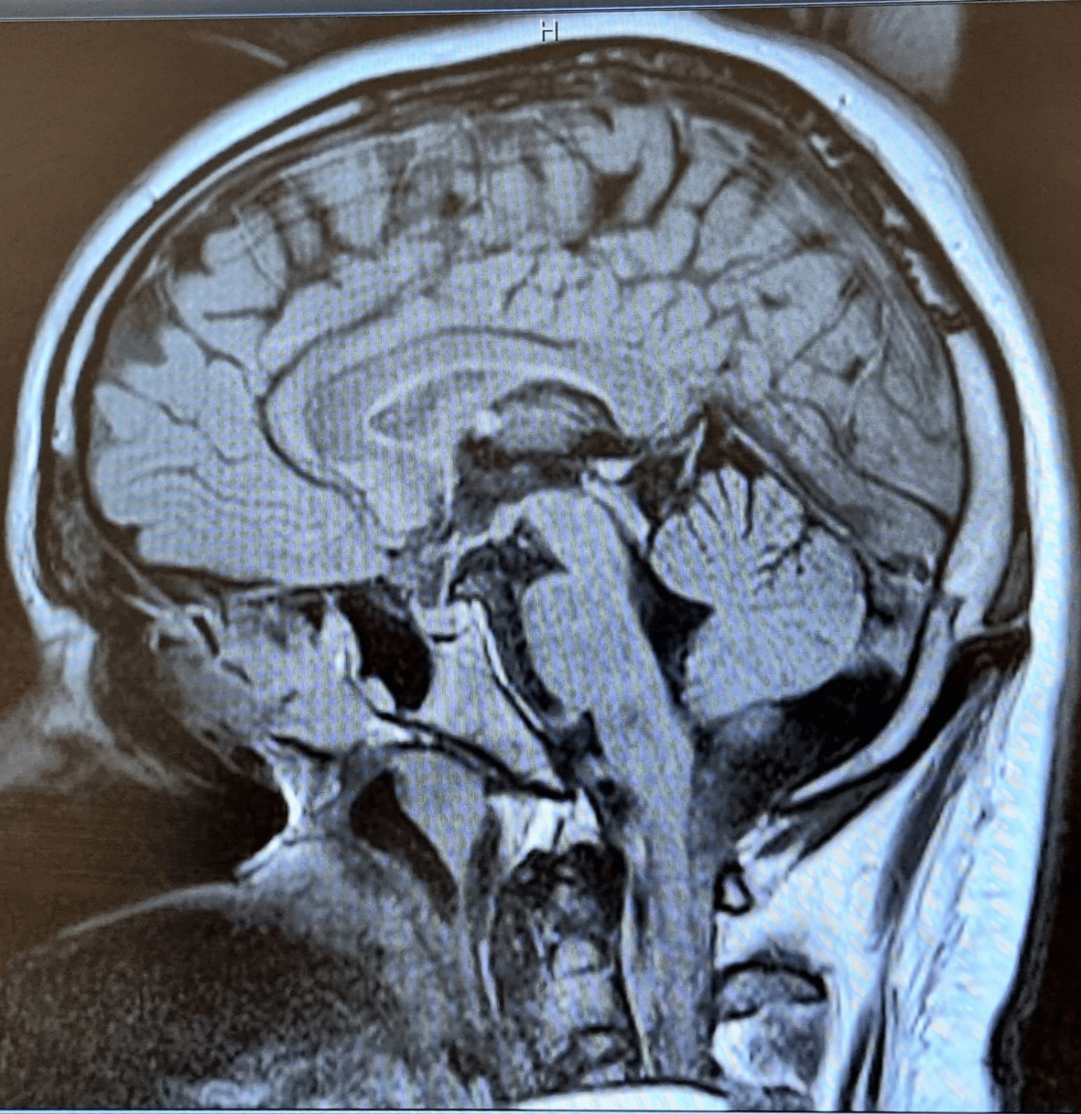
The MRI of Case 1's brain (sagittal T1 image) shows no gross pathology.

Radiological investigations, including chest and abdominal X-rays, were unremarkable, showing no significant abnormalities. Visual evoked potential (VEP) showed mild conduction defects in the anterior visual pathway of the left optic nerve (Figure 1). The P100 latency, which is the time from the stimulus onset to the main positive peak, was prolonged, driven mainly by pre-chiasmatic lesions. Prolonged P100 latency usually represents an optic nerve dysfunction. An MRI of the brain also showed no evidence of gross abnormalities (Figures 2, 3). An abdominal ultrasound revealed cholelithiasis without any common bile duct stone or dilation. The nerve conduction study was normal, ruling out peripheral neuropathy. In summary, the investigations pointed towards a significant thiamine deficiency, likely contributing to the patient's clinical presentation, alongside evidence of gastritis, duodenitis, and cholelithiasis, without significant liver dysfunction or peripheral neuropathy. The elevated CRP levels and the presence of ketones in the urine suggest an acute inflammatory process and metabolic stress, respectively. The patient began treatment with thiamine. Betahistine was given to address dizziness. He received supportive treatment with IV hydration, proton pump inhibitors (PPIs), paracetamol, and ondansetron as necessary. Improvements in the patient's neurological signs and symptoms were noted with thiamine supplementation after three days, and he was discharged in baseline condition. The patient was seen by a dietitian and was advised to maintain a healthy, well-balanced diet with increased thiamine-rich foods and decreased carbohydrate intake, aiming to prevent recurrence.

Case 2

An 18-year-old male patient presented with a two-week history of recurrent non-projectile vomiting following meals, uncontrolled hypertension, and significant weight loss of 25 kg over two to three weeks. He also reported a previous mild COVID-19 infection a month prior. The condition coincided with his recent initiation into strenuous exercise routines. Two days before the presentation, he experienced generalized weakness, blurred vision in the left eye, a left-sided headache, and dizziness. Subsequently, the next day, visual obscuration worsened to involve both eyes, accompanied by a mild headache and double vision that improved upon closing the left eye. This visual impairment led to difficulties in walking. Additionally, he reported a day of tinnitus. The patient denied any use of illicit drugs or alcohol.

The patient's vital signs were stable, with a body temperature fluctuating between 36.5°C and 37.2°C, averaging at 36.9°C orally. The heart rate was slightly elevated at 96 beats per minute, while the respiratory rate was normal at 16 breaths per minute. Blood pressure was within normal range at 127/74 mmHg, and oxygen saturation was optimal at 98%. The patient displayed mild signs of dehydration. Ocular examination showed impaired visual acuity and color vision bilaterally, limited abduction of the right eye, bidirectional gaze evoked horizontal nystagmus, and fundoscopy confirmed the presence of papilledema bilaterally. No gait abnormalities were observed. The rest of the neurological exam was unremarkable.

Investigations upon admission revealed a normal hemoglobin (Hb) level and platelet count. Creatinine level was normal, indicating normal kidney function. The liver function tests showed high total bilirubin, direct bilirubin, and ALT with almost normal AST and ALP, suggesting a degree of liver stress or damage. He also underwent a lumbar puncture. The cerebrospinal fluid (CSF) analysis was normal, and oligoclonal bands were negative. Serum aquaporin-4 antibodies (AQP4-Ab) were negative. The patient's laboratory results are summarized in Table 2.

**Table 2:** A summary of Case 2's laboratory results Hb: hemoglobin; AST: aspartate transaminase; ALT: alanine transaminase; ALP: alkaline phosphatase µmol/L (micromoles per liter), g/dL (grams per deciliter), mg/dL (milligrams per deciliter), and U/L (units per liter) are standard measurement units.

Test	Result	Normal range	Notes
Hb	13.1 g/dL	13-16 g/dL	Normal
Platelet count	321,000/µL	150,000-450,000/µL	Normal
Creatinine	62 µmol/L	61.9-114.9 µmol/L	Normal
Total bilirubin	17.2 mg/dl	0.1 - 1.2 mg/dL	Elevated
Direct bilirubin	6.4 mg/dl	<0.3 mg/dl	Elevated
AST	35 U/L	8-33 U/L	Slightly Elevated
ALT	101 U/L	4-36 U/L	Elevated
ALP	71 U/L	44-147 U/L	Normal

**Figure 4:**
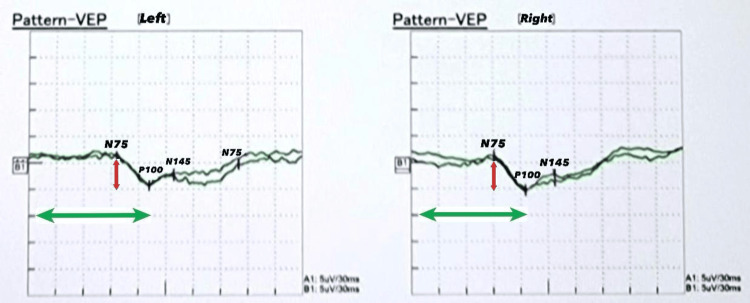
The VEP of Case 2 shows a distorted waveform with moderately prolonged P100 latencies more on the left side and normal amplitude bilaterally, denoting moderate bilateral visual pathway dysfunction more pronounced on the left side. VEP: visual evoked potential

**Figure 5:**
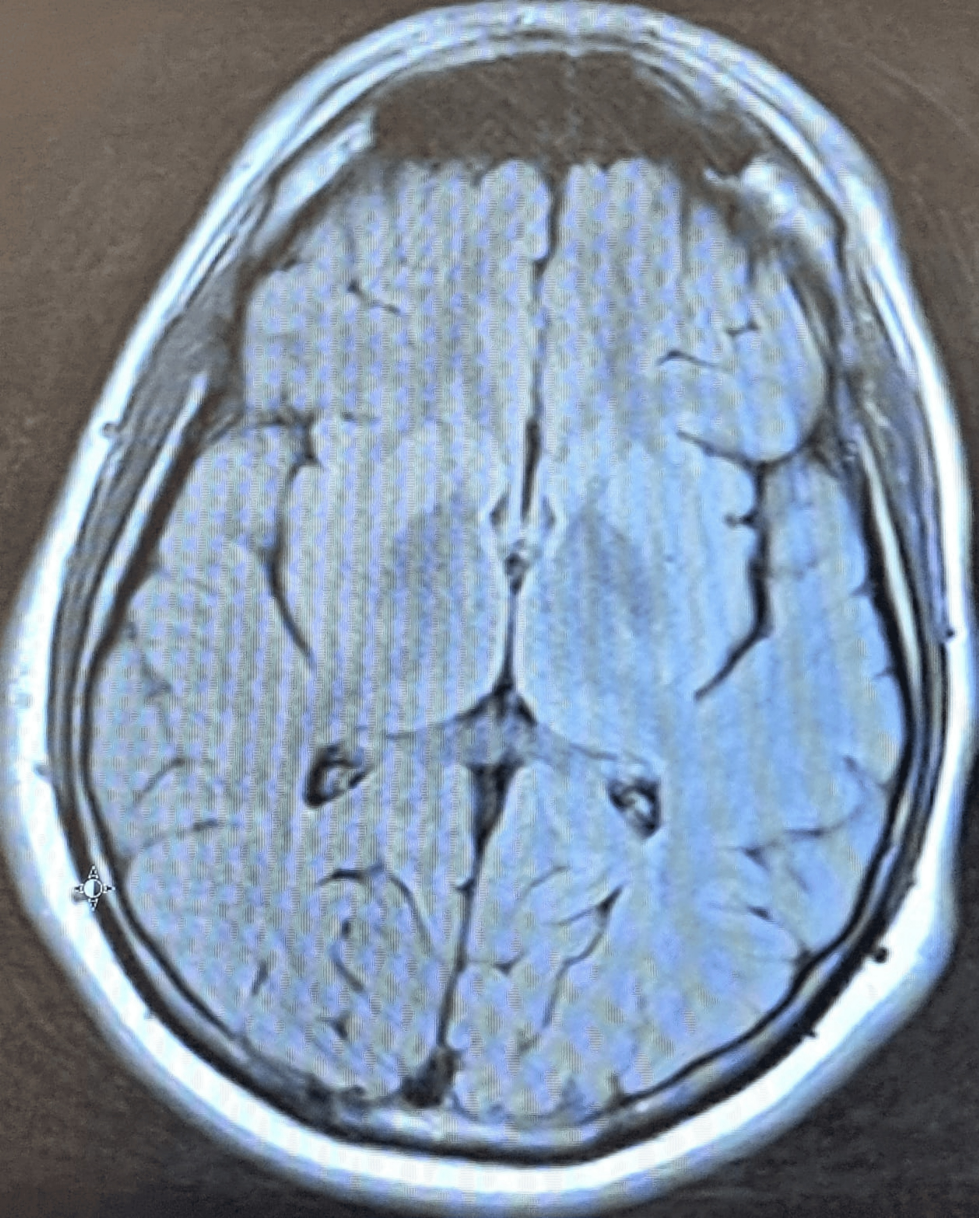
The MRI of Case 2's brain (axial FLAIR image) shows no gross pathology. FLAIR: fluid-attenuated inversion recovery

Neurophysiological tests included a VEP, indicating moderate bilateral visual pathway dysfunction, with a more pronounced impact on the left (Figure 4). The nerve conduction study was normal. An MRI of the brain showed no evidence of gross abnormalities (Figure 5). He also underwent further testing for indirect hyperbilirubinemia. Ultrasound of the abdomen showed a sludge within the gallbladder, and no calculus was detected. He was diagnosed by exclusion as having Gilbert syndrome. The diagnosis was established as probable thiamine deficiency secondary to recurrent vomiting, given the constellation of neurological and ocular findings. The patient was admitted, and IV thiamine was administered along with symptomatic measures, resulting in significant recovery. Due to the patient's prior empirical treatment with thiamine injections, thiamine levels were not measured. Other possible differential diagnoses included Miller Fisher syndrome and demyelinating diseases, which were considered initially due to the acute presentation of ocular manifestations and gait difficulty. Deep tendon reflexes were intact, and the NCS study was normal, excluding Miller Fisher syndrome. The MRI of the brain came back normal, making the diagnosis of demyelinating diseases unlikely. Considering the clinical presentation and the absence of significant findings in other laboratory tests, thiamine deficiency emerged as the most likely diagnosis.

Significant improvements in the patient's neurological signs and symptoms were noted with thiamine supplementation after one week. Dietitian advice was given, aiming to prevent recurrence. The blurring of vision and diplopia resolved completely, and he was discharged. He was seen in the outpatient clinic two weeks later; he was still having gaze-evoked nystagmus, and the patient's gait improved.

Case 3

An 18-year-old male patient presented with a three-day history of sudden-onset diplopia and blurred vision. He described a two-week history of recurrent vomiting and a significant weight loss of 25 kg over the past nine weeks. He went on an unbalanced diet with very low calories and consumed a high-carbohydrate diet, which coincided with his recent initiation into strenuous exercise routines. He had no relevant past medical or family history. The patient denied any use of illicit drugs or alcohol.

During the clinical examination, the patient's vital signs were within normal limits. Neurological examination showed bilateral rotatory nystagmus and a horizontal gaze, yet the fundoscopy was normal. Visual assessments, including acuity, color vision, and visual fields, were all normal. There was no evidence of facial drooping, and the patient's gait was normal too. Further neurological assessments, encompassing sensation, strength, coordination, reflexes, and an examination of the cranial nerves apart from the eye findings previously mentioned, revealed no abnormalities.

Blood tests, including a complete blood count (CBC) and a comprehensive metabolic panel (CMP), were all found to be within normal limits. Thiamine levels were assessed after administering thiamine replacement, resulting in elevated vitamin B1 levels, following a single intravenous dose of vitamin B1. This measurement, however, did not accurately reflect the patient's baseline thiamine status due to the timing of the assessment post-supplementation. Additionally, decreased transketolase activity was observed, alongside a normal TPP. These findings suggested altered thiamine metabolism, which, despite the high serum vitamin B1 levels, may indicate a functional deficiency. Aquaporin-4 antibodies returned negative. The patient's laboratory results are summarized in Table 3.

**Table 3:** A summary of Case 3's laboratory results TPP: thiamine pyrophosphate ng/L (nanograms per liter), U/L (units per liter), and ng/mL (nanograms per milliliter) are standard measurement units.

Test	Result	Normal range	Notes
Thiamine (vitamin B1)	122 ng/L	70-180 ng/L	Normal
Transketolase activity	29.2 U/L	42-100 U/L	Below normal
TPP effect	5.6 ng/mL	< 20 ng/mL	Normal

**Figure 6:**
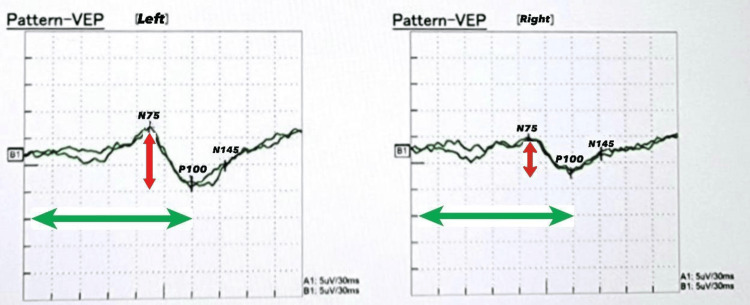
The VEP of Case 3 shows a distorted waveform with severely prolonged P100 latencies bilaterally and reduced amplitude notes from the right side, denoting severe bilateral visual pathway dysfunction.

**Figure 7:**
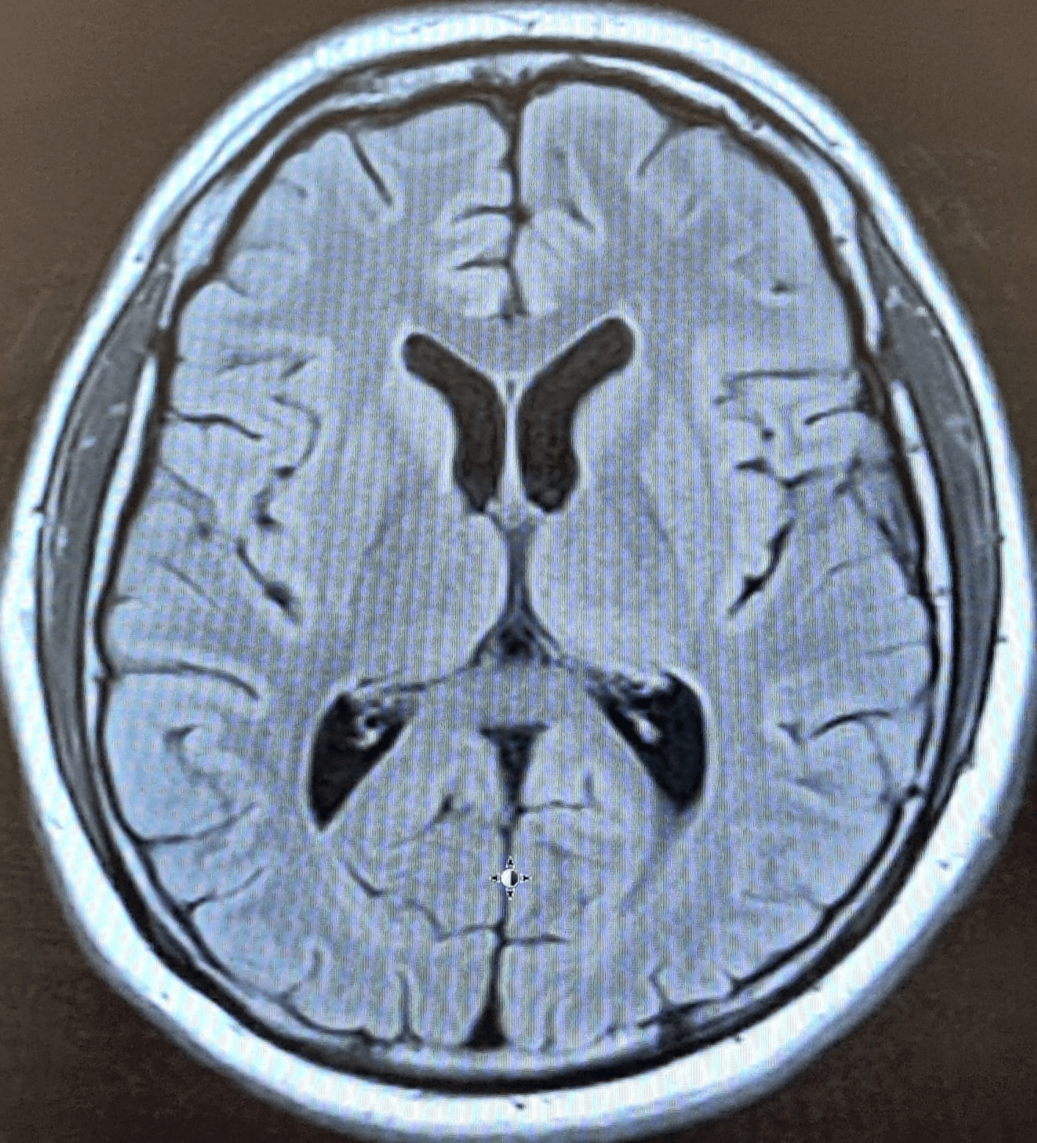
The MRI of Case 3's brain (axial FLAIR image) shows no gross pathology.

**Figure 8:**
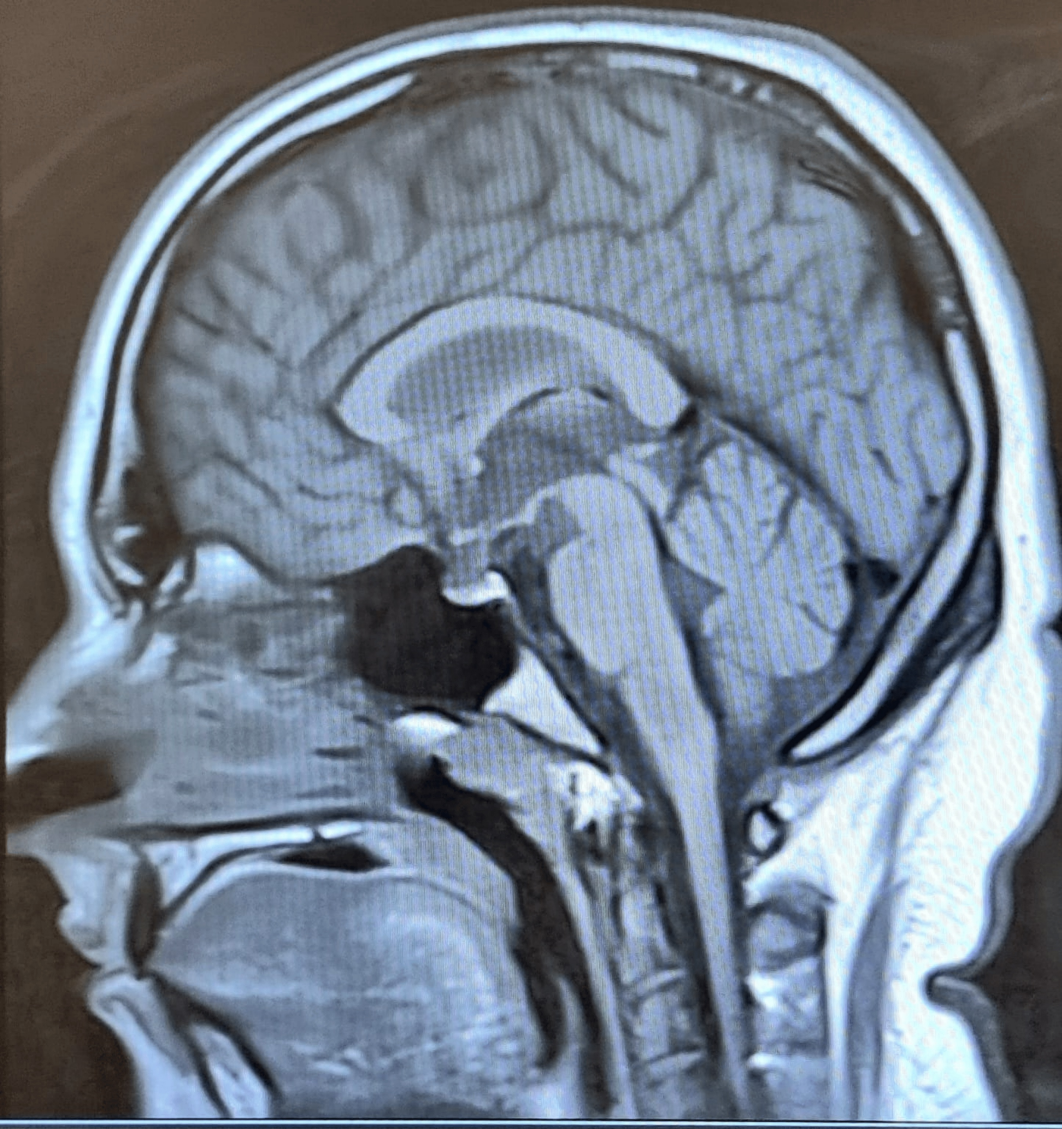
The MRI of Case 3's brain (sagittal T1 image) shows no gross pathology.

The VEP demonstrated prolonged P100 latencies on both sides. This specific finding indicated a delay in the neural conduction time within the visual pathways (Figure 6). Both CT and MRI scans of the brain revealed no significant abnormalities (Figures 7, 8). A diagnosis of thiamine deficiency was made, primarily linked to the recurrent episodes of vomiting. The swift weight loss, compounded by the recent bouts of vomiting, likely intensified the vitamin B1 deficiency. This was further evidenced by eye movement abnormality and the neurophysiological indications of optic involvement. Improvements in the patient's neurological signs and symptoms were noted with thiamine supplementation after one week. Dietitian's advice was given, aiming to prevent recurrence. Upon discharge, his eye movements were normal with only horizontal nystagmus remaining. The patient didn’t visit the outpatient clinic after discharge.

## Discussion

Thiamine deficiency affects all the body's systems, but especially the brain and heart. Thiamine deficiency has a greater impact on the central nervous system because it is so reliant on glucose for energy. It affects both glial cells and neurons in the brain [7]. Thiamine deficiency presents with vague symptoms such as fatigue, irritability, poor memory, appetite loss, poor sleep, abdominal discomfort, and loss of weight. This complicates its diagnosis. Wernicke's encephalopathy, which includes an acute confusional state, ataxia, and internuclear ophthalmoplegia, is the most classic presentation of thiamine deficiency [2]. Up to 20% of critically ill patients have a thiamine deficiency [1]. The thiamine requirement of the body is determined by its metabolic demand. Infection and sepsis can cause thiamine deficiency. A healthy person can become deficient in thiamine after a dietary disruption of about three weeks [8].

With thiamine deficiency, optic neuropathy is rare [9]. Visual acuity and color vision can be affected and improved by taking thiamine. Similar to case two in our study, visual impairment due to optic disc swelling has also been reported. Some studies mention optic disc pale edema and retinal hemorrhages [9-11]. According to van Noort et al., the optic neuropathy in Wernicke's encephalopathy is caused by hemorrhages in the optic nerve and the nerve fiber layer of the retina. By affecting the vestibular nuclei, these hemorrhages can alter the oculomotor system and cause nystagmus [11].

A case series of three patients with ophthalmological symptoms and a history of vomiting and weight loss for weeks was presented. Consistent vomiting depletes the body of thiamine and contributes to weight loss. Thiamine deficiency manifested as nystagmus, diplopia, and blurred vision in our patients. Following the administration of thiamine, these symptoms improved.

The MRI of the brain didn’t show any gross abnormalities in our three cases. An MRI is not highly sensitive in detecting early-stage thiamine deficiency. MRI abnormalities, such as hyperintensities in the mammillary bodies, thalamus, periaqueductal gray, and tectal plate, often appear in moderate to advanced stages, and these changes are nonspecific [3-7]. This makes MRI less reliable as a sole diagnostic tool. Hence, overly relying on imaging results instead of a strong clinical indication may delay treatment.

Thiamine deficiency, while a grave medical concern, often presents itself in myriad ways. The involvement of the optic nerve, whether through optic neuritis or papilledema, stands out as a particularly uncommon presentation. This uncommon presentation can be misleading for practitioners, potentially leading to misdiagnoses or delayed treatment. It is vital to be vigilant and consider thiamine deficiency as a differential diagnosis in cases of unexplained optic nerve involvement. Also, clinicians must consider exercise habits and other potential stressors when evaluating patients with potential thiamine deficiency symptoms. Given the severe consequences of untreated thiamine deficiency, early detection and swift treatment are paramount. Thiamine levels can rise quickly after supplementation, similar to case three in our study; this can give a false impression of sufficiency when deficiency-related symptoms persist, highlighting the importance of relying on clinical presentation for diagnosis. Thiamine deficiency is a clinical emergency that can progress to irreversible brain damage. Empirical thiamine administration is safe, inexpensive, critical to preventing complications, and a response to thiamine therapy strongly supports a deficiency. A high index of clinical suspicion can make the difference between recovery and lasting neurological damage. 

## Conclusions

Non-alcoholic thiamine deficiency with rare neuro-ophthalmic complications should be kept in mind as a differential diagnosis in cases of unexplained optic nerve involvement. Healthcare professionals must recognize these critical aspects of thiamine deficiency and its atypical presentation. A heightened level of clinical susception can be the key factor in distinguishing between recovery and permanent neurological damage. Excessive reliance on imaging findings, rather than prioritizing strong clinical indications may delay treatment initiation. Being equipped with this knowledge can significantly impact patient outcomes, ensuring timely intervention and preventing irreversible complications.
